# Supplementary material for: Structural basis of transcription arrest by coliphage HK022 Nun in an Escherichia coli RNA polymerase elongation complex
Source: eLife. 2017 Mar 20;6:e25478. doi: 10.7554/eLife.25478 (PMC5386594; doi:10.7554/eLife.25478)
Supplement: Supplementary file 1. — DOI: http://dx.doi.org/10.7554/eLife.25478.023 [file elife-25478-supp1.docx]

**Supplementary file 1. Model statistics from Molprobity** (Chen et al., 2010)**.**

|  | X-Nun/TEC | X-TEC | TEC |
| --- | --- | --- | --- |
| Resolution^a^ | 3.74 Å | 4.05 Å | 4.38 Å |
| Molprobity score | 2.29 | 2.42 | 2.45 |
| Clashscore (all atoms) | 17.12 | 20.75 | 24.14 |
| Rotamer outliers | 0.07% | 0.07% | 0.38% |
| RMS deviations bonds (Å) | 0.01 | 0.01 | 0.01 |
| RMS deviations angles (°) | 0.95 | 0.88 | 0.96 |
| Ramachandran favored | 90% | 88% | 89% |
| Ramachandran outliers | 0.34% | 0.41% | 0.41% |

^a^ Gold standard FSC 0.143 cutoff criteria (Figure 1 – figure supplements 2F, 3E, 4D; (Rosenthal and Henderson, 2003).
