# Supplementary material for: Structural basis of transcription arrest by coliphage HK022 Nun in an Escherichia coli RNA polymerase elongation complex
Source: eLife. 2017 Mar 20;6:e25478. doi: 10.7554/eLife.25478 (PMC5386594; doi:10.7554/eLife.25478)
Supplement: Supplementary file 2. — DOI: http://dx.doi.org/10.7554/eLife.25478.024 [file elife-25478-supp2.docx]

**Supplementary file 2. Results of superpositions of TEC models.**

|  | X-TEC^a^ | X-Nun/TEC^b^ |
| --- | --- | --- |
| TEC^c^ | 0.646 Å^d^  (2,750 Cα’s)^e^ | 0.689 Å^d^  (2,838 Cα’s)^e^ |
| X-TEC |  | 0.698 Å^d^  (2,806 Cα’s)^e^ |

^a^ Gluteraldehyde crosslinked TEC-20A model (refined at 4.05 Å resolution).

^b^ Gluteraldehyde crosslinked Nun/TEC-20A model (refined at 3.74 Å resolution).

^c^ Uncrosslinked TEC-20A model (refined at 4.38 Å resolution).

^d^ Root mean square deviation of Cα positions, calculated using the ‘align’ command in PyMOL (The PyMOL Molecular Graphics System, Version 1.6 Schrödinger, LLC).

^e^ Number of Cα positions aligned (out of 3,198 RNAP residues).
